# Assessment of swallowing performance in patients with neurodegenerative disease: A hierarchical cluster analysis

**DOI:** 10.1002/brb3.70005

**Published:** 2024-08-28

**Authors:** Samet Tosun, Fenise Selin Karali, Dilber Kacar Kutukcu, Nilgün Cinar, Sude Kendirli, Meltem Sen Aksut, Ilayda Albayrak, Yusuf Celik

**Affiliations:** ^1^ Faculty of Health Sciences, Department of Speech and Language Therapy Biruni University Istanbul Turkey; ^2^ School of Medicine, Department of Neurology Maltepe University Istanbul Turkey; ^3^ School of Medicine, Department of Biostatistics and Medical Informatics Biruni University Istanbul Turkey

**Keywords:** dementia, dysphagia, neurodegenerative diseases, swallowing

## Abstract

**Background:**

Swallowing is a complex process that alters with age and neurological diseases; swallowing disorders can be a consequence of both of them. As an advanced multivariate statistical method, hierarchical cluster analysis (HCA) was utilized to make the dendrograms, which was used to find the relationship between the variables. The purpose of this study is to ascertain the type of clustering exhibited by the variables using HCA and to evaluate the approach to major neurodegenerative diseases (MND) with swallowing disorders based on the results obtained.

**Methods:**

Data were collected from a total of 173 patients from various neurological diagnoses, such as dementia, Parkinson's disease, stroke and polyneuropathy, aging between 42 and 104 (mean of age 72.85) by using the Montreal Cognitive Assessment, the Edinburgh Feeding Evaluation Scale (EdFED), the Eating Assessment Tool (EAT‐10), and the Modified Mann Swallowing Ability test. From the collected data, dendrograms were formed by using HCA with Ward linkage method.

**Results:**

Based on cluster analysis results, clusters demonstrate statistical significance. They center around EdFED, EAT‐10, and age in each MND. In healthy individuals, variables are not clustered as in the patient group. This study holds importance as it can give clinicians a different perspective on determining and managing the elderly population's swallowing problems.

**Conclusions:**

The HCA method explicitly proposes which variables should be examined concurrently in the clinic for MND. This research is one of the pioneering studies conducted by using the HCA method.

## Graphical abstract

This study uses hierarchical cluster analysis (HCA) to examine the relationship between variables in major neurodegenerative diseases (MND) with swallowing disorders. Data were collected from 173 patients with neurological diagnoses, aging between 42 and 104. The results showed statistical significance in clusters around Edinburgh Feeding Evaluation Scale (EdFED), Eating Assessment Tool (EAT‐10), and age in each MND. This study provides clinicians with a different perspective on determining and managing elderly swallowing problems, suggesting that variables should be examined concurrently in clinics for MND.

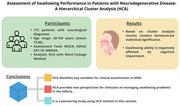

## 1 INTRODUCTION

The number of elderly individuals in the world's population is rising steadily, and with it comes an associated rise in the incidence of neurodegenerative diseases (Lin et al., 2008). Patients suffering from neurodegenerative disorders not only encounter cognitive disorders but also experience swallowing difficulties (Watson & Green, 2006). The problems associated with food, neurodegenerative diseases, and feeding have received considerable attention by speech and language therapist (SLT).

Dementia is one of the most common neurogenic diseases associated with aging. It is a condition in which cognition gradually deteriorates, affecting daily functions. Multiple cognitive abilities are impaired in a person with dementia, most frequently memory, but also language, attention, orientation, judgment, and planning (Arvanitakis & Bennett, 2019). Alzheimer's disease (AD) and other dementia‐causing illnesses are progressive and incurable, resulting in a complete loss of cognitive and bodily functions. Loss of interest in eating, dysphagia, or both are common symptoms of the early and final stage of dementia, which can last from 6 months to 2 years (Goldberg & Altman, 2014).

Dysphagia is a frequent sign of dementia and other major neurodegenerative diseases. It is estimated that up to 45% of institutionalized dementia patients have some degree of difficulty swallowing (Easterling & Robbins, 2008). Dysphagia refers to swallowing difficulties caused by oropharyngeal or esophageal dysfunction (Alagiakrishnan et al., 2012). Distinct clinical manifestations of dementia are associated with various swallowing and feeding impairments (Sura et al., 2012). Patients with dementia typically exhibit a slowing of the swallowing process. Slowed swallowing processes may lengthen the time required to complete a meal, thereby increasing the risk of poor nutritional status (Groher & Crary, 2020; Morley, 2002). Moreover, patients with dementia frequently struggle with self‐feeding (Chen et al., 2016). These difficulties may stem from cognitive impairment, motor deficits such as weakness or apraxia, appetite loss, and/or food aversion food aversion (Lewis et al., 2018). As a result, dementia patients may experience weight loss and an increased reliance on feeding (Easterling & Robbins, 2008).

In the early stages of AD, dysphagia is characterized by a prolonged oral stage marked by reduced lingual movement and a delayed swallowing reflex (Sato et al., 2014). This prolonged oral phase has been associated with a longer meal duration and, consequently, an increased risk of malnutrition (Suto et al., 2014). Moderate stages of AD are characterized by difficulties in bolus preparation, airway clearance, upper esophageal sphincter opening, and visible aspiration (Alagiakrishnan et al., 2013). In the severe stage, swallowing difficulties are severe and have a substantial impact on the individual's quality of life.

In order to understand the relationship between major neurodegenerative diseases (MND) and swallowing disorders better, a new approach should be taken. In this study, we decided to use a new method known as hierarchical cluster analysis (HCA). HCA is a prominent cluster analysis technique in data research and data mining that aims to establish a hierarchy of clusters. HCA attempts to cluster subjects with similar characteristics (Murtagh, 2014). HCA employs two categories of strategies: the agglomerative strategy and the divisive strategy. With agglomerative clustering proceeding from “the leaves” to “the root” of a cluster tree, this method is referred to as a “bottom up” method. Divisive clustering is a “top‐down” technique that works from the roots to the leaves. Initial consideration of all observations as a single cluster is followed by recursive divisions as one descends the hierarchy (Gil‐Garcia et al., 2006). Using an algorithm that begins with each variable in a separate cluster and combines clusters until only one remains, this procedure attempts to identify relatively homogeneous groups of variables based on selected characteristics. Using HCA, we are able to determine which variables are most closely associated with one another and their differences.

As it is known that SLTs play a crucial role in the assessment and treatment of dysphagia, collaborating with a variety of health professionals (Clark & Ebersole, 2018). In diagnosing dysphagia, screening, physical examination, and supplementary tests all play an important role. Several dysphagia screening tools such as the Eating Assessment Tool (EAT‐10) (Özsürekci et al., 2020), the Turkish Modified Mann Assessment of Swallowing Ability (TR‐MMASA) test (Ciftci & Topbas, 2022), and the Edinburgh Feeding Evaluation in Dementia (EdFED) (Uyar et al., 2022) have been used.

Although each of these tests provides distinct information about swallowing disorders and cognition, the results of all of these tests should be considered and evaluated collectively when diagnosing and treating swallowing disorders. However, there is no study in the literature that evaluates all of these studies conjointly and adjusts clinical practice accordingly. The purpose of this study is to ascertain the type of clustering exhibited by the variables using HCA and to evaluate the approach to MND with swallowing disorders based on the results obtained. In light of these factors, we seek answers to the following research questions:

1. Is HCA capable of revealing the relationships among evaluation instruments?
2. Which of the variables (age and education) are clustered with measurement tools in HCA?
3. Is it possible to reveal relationships between cognitive and swallowing disorders by using HCA?

## 2 METHODS

This is a cross‐sectional and descriptive study. This study received ethical clearance from the Ethics Committee of Biruni University University (Protocol Number: 2015‐KAEK‐72‐22‐05). Patients who applied to Maltepe University University Neurology Clinic were enrolled in this study. Written consent form was obtained from each participant or patient's caregiver. Relevant information was taken from each patient or patients’ caregiver in a session lasting about 30 min to 1 h.

### 2.1 Participants

This research was carried out with the participation of 173 individuals in total. The participants' demographic information is compiled and documented in Table 1.

**TABLE 1:** Demographic information.

			Gender		Education				
Group	Number of participants	Mean of age	Female	Male	Illiterate	Primary school	Secondary school	High school	University
Alzheimer's disease	34	79.79 ± 7.62	24	10	1	18	1	4	10
Parkinson's disease	28	74.63 ± 9.85	18	10	1	12	0	7	8
Stroke	26	72.53 ± 9.18	10	16	0	10	2	5	9
Polyneuropathy	15	70.46 ± 10.06	10	5	2	5	0	6	2
Other dementia types	43	72.53 ± 10.37	30	13	1	20	5	8	9
Control group	27	64.40 ± 4.67	16	11	0	10	1	8	8
Total	173	72.85 ± 9.91	108	65	5	75	9	38	46

Throughout the investigation, the convenience sampling method was used; data were gathered from the same neurology clinic and the patients were diagnosed by the same neurologist over the study period.

While diagnosing dementia patients, the diagnostic criteria established by the National Institute of Neurological and Communicative Disorders and Stroke‐AD and Related Disorders Association was used (McKhann et al., 1984).

The inclusion criteria for MND patients were as follows: (1) having been diagnosed with MND, (2) having adequate sensory acuity to complete the tasks, (3) not having had head trauma in the last 2 years, and (4) being native Turkish speakers. The exclusion criteria for dementia patients were as follows: (1) being unable to complete the study, (2) not having adequate sensory acuity to complete the tasks, and (3) having history of psychiatric disease.

The inclusion criteria for the healthy control group were as follows: (1) accepted as healthy after comprehensive examination by a neurologist, (2) having adequate sensory acuity to complete the tasks, (3) not having any visual or hearing impairment, and (4) being native Turkish speaker. The exclusion criteria for the healthy control group were as follows: (1) being unable to complete the assessment tools, (2) withdrawing from the study, and (3) having history of psychiatric disease. All of our patients were outpatients who applied to the neurology clinic; nevertheless, we had to exclude a few of them for various reasons, such as failing to provide consent (seven patients) or refusing to finish tasks (12 patients). Therefore, we had 173 patients in total.

### 2.2 Data collection tools

Among the data collection tools used in this study are sociodemographic information form, the Montreal Cognitive Assessment (MoCA) scale (Nasreddine et al., 2005; Selekler et al., 2010), the Eating Asessment Tool (EAT‐10) (Belafsky et al., 2008; Demir et al., 2016), the TR‐MMASA test (Ciftci & Topbas, 2022), and the EdFED (Uyar et al., 2022; Watson et al., 2001; Watson & Dreary, 1997). Detailed information about these tools is given below.

#### 2.2.1 Sociodemographic information form

By using this form, the participants' medical histories, diagnoses, and their demographic information such as age and gender data were collected.

#### 2.2.2 The Montreal Cognitive Assessment scale

It was formed as a quick screening test for mild cognitive impairments. It measures different cognitive functions. Attention and concentration, executive functions, memory, language, visual construction skills, abstract thinking, calculation, and orientation are some of these cognitive functions (Nasreddine et al., 2005; Selekler et al., 2010).

#### 2.2.3 Swallowing Function Screening Test (EAT‐10)

It is a test used to screen patients at risk to determine the severity of symptoms and to evaluate the effectiveness of the ongoing treatment. It can be used in adults with neurological conditions (such as Alzheimer's and Parkinson's disease [PD], etc.), who are weak, dependent on social services, living in nursing homes or care centers, or elderly patients with pneumonia cases (Belafsky et al., 2008; Demir et al., 2016).

#### 2.2.4 Turkish Modified Mann Swallowing Assessment test

TR‐MMASA is a shortened version of MASA, which is a more complex assessment tool that can be performed at the bedside in less than 5 min. The TR‐MMASA test has also been used to assess swallowing function in patients with mild to moderate dementia who are not suffering from an acute stroke (Ciftci & Topbas, 2022). This test should be applied by an SLT, and it was applied accordingly in this study.

#### 2.2.5 Edinburgh Feeding Evaluation in Dementia

The EdFED scale, developed by Watson and Dreary (1997), measures feeding difficulties in elderly individuals with dementia. The scale that quickly reveals swallowing problems is a practical, reliable, and valid instrument. Dementia is associated with impaired functionality and disability and has a high physical, psychological, social, and economic impact on patients and caregivers. Assessment of decreased functionality and disability can be time consuming. One of these disabilities is swallowing disorders. The EdFED (Watson & Dreary, 1997) is an 11‐point feeding evaluation scale. Use of this scale makes suspicion of dysphagia clear. Therefore, the EdFED can be used to prevent clinicians from missing out on any swallowing disorder presence (Uyar et al., 2022; Watson et al., 2001; Watson & Dreary, 1997).

### 2.3 Procedure

After receiving approval from the ethics committee, data were collected from patients who applied to the Maltepe University University neurology clinic by using the sociodemographic information form, MoCA, the EAT‐10, the TR‐MMASA, and the EdFED scales. After completing a full neurological examination, patients diagnosed with neurological problems were referred to SLT for further evaluation. The cognitive and swallowing performance of the patients were measured. Finally, with these data, a statistical analysis was performed by using HCA.

### 2.4 Data analysis

HCA is one of the latest multivariate statistical methods. It was used to figure out how the variables in our study tend to group together. A dendrogram showed how similar things were based on how they related to the variables. The Common Linkage and Ward's Hierarchical Clustering model were used to make the dendrogram of the variables. The chosen model should be the best one based on how the data are organized, with as little variation within clusters as possible and as much variation as possible between clusters. So, the conclusions from this method are stronger than those from the univariate methods. Each variable for two clusters was analyzed by Kruskal–Wallis test. Two‐sided *p* values were considered statistically significant at *p* ≤ .05. Statistical analyses were performed using R software/programming (version 3.6.2 (2019‐12‐12)—CRAN).

## 3 RESULTS

A total of 173 participants took part in our study. Patients with neurological disorders who were referred for an SLT assessment after a comprehensive neurological examination (*n* = 173; 65 males and 108 females, average age 72.85 ± 9.91 years) and 27 healthy controls (average age 64.4 ± 4.68 years) were included in our study. These patients were classified into five categories based on their diagnosis: 34 cases with AD, 28 patients with PD, 26 patients with stroke, 15 patients with polyneuropathy, and 43 patients with other types of dementia. Using HCA of multivariate statistical methods, the variables' tendency to group together was shown in a dendrogram, which was used to figure out how these groups can come together.

**FIGURE 1:**
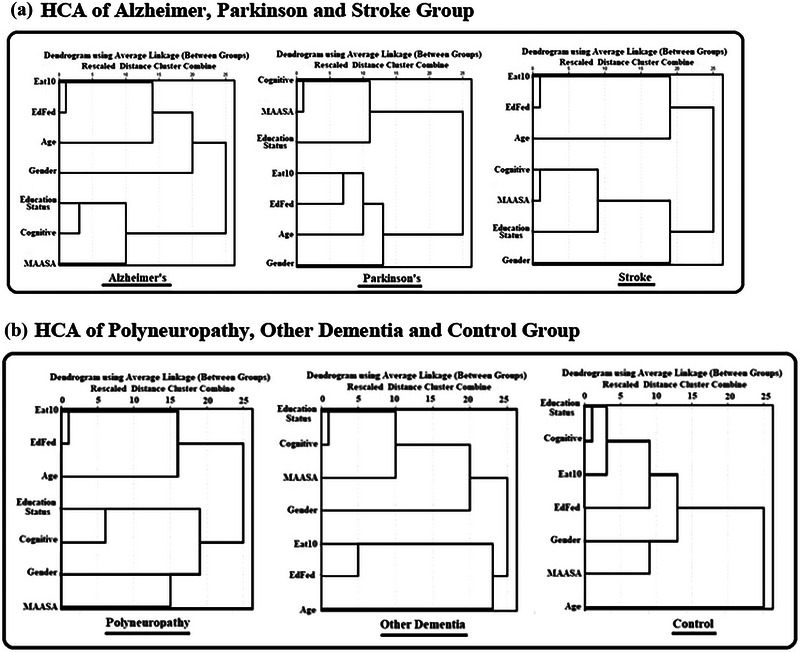
(a) Hierarchical cluster analysis (HCA) of Alzheimer, Parkinson, and stroke group. (b) HCA of polyneuropathy, other dementia, and control group.

Results of the cluster analysis are displayed graphically in Figure 1a,b. Figure 1a shows the HCA for Alzheimer, Parkinson, and stroke patients. Figure 1a shows that the HCA for Alzheimer's patients two main clusters were obtained. Main cluster I contains EAT‐10, EdFED, age, and gender; and main cluster II contains education status, cognitive function, and TR‐MMASA. As seen in Figure 1a, which showed the HCA for Parkinsons's patients, two main clusters were obtained. Main cluster I contains cognitive function, TR‐MMASA, and education status; and main cluster II contains EAT‐10, EdFED, age, and gender. One of the important points is that the HCA obtained two main clusters for stroke patients. Main cluster I contains EAT‐10, EdFED, and age; and main Cluster II contains cognitive function, TR‐MMASA, education, and gender.

Figure 1b depicts that the HCA for polyneuropathy patients obtained two main clusters. Main cluster I contains EAT‐10, EdFED, and age; and main cluster II contains education status, cognitive function, gender, and TR‐MMASA. As it could be described in Figure 1b, the HCA obtained two main clusters for other types of dementia. Main cluster I contains education status, cognitive function, TR‐MMASA, and gender; and main cluster II contains EAT‐10, EdFED, and age. As shown in Figure 1b, the HCA obtained two clusters for the control group. Main cluster I contains education status, cognitive function, EAT‐10, EdFED, gender, and TR‐MMASA; and main cluster II contains age. In Figure 1a, gender variable is closely related to EAT‐10, EdFED, and age in AD. This is only seen in this population. It is noteworthy that the clustering of variables in the control group was also properly correlated. In other words, when there is no disease, clustering does not occur.

## 4 DISCUSSION

In this study, we investigated the swallowing performance and severity of swallowing disorders in 173 participants. HCA method could provide clinicians with a novel method when they need to take assessment of a patient into consideration. HCA selects the clusters that are the most closely related to one another and then merges the two clusters that are the most comparable.

Based on the literature, oropharyngeal dysphagia is a common complication of stroke, PD, and AD, and can lead to malnutrition, aspiration pneumonia, and early death. Dysphagia is frequently neglected and underdiagnosed in vulnerable patient populations, despite its high frequency among the elderly (Takizawa et al., 2016).

When investigated, in each subcluster EAT‐10 and EdFED were grouped together, they both are used to assist clinicians in identifying eating and feeding issues in patients (Belafsky et al., 2008; Lin et al., 2008). Even though EdFED is only utilized to determine eating problems in individuals with late‐stage dementia, our study reveals clinicians can use EdFED to measure swallowing performance of all types of MND. Both EAT‐10 and EdFED are short and cost‐effective tools in clinical practice and their Turkish versions are also valid and reliable (Demir et al., 2016; Uyar et al., 2022). It can be clearly seen as in figures, EAT‐10 and EdFED are placed in the same cluster, which shows either EAT‐10 or EdFED could be utilized by clinicians. It would be wise for clinicians to use the tool with which they are more familiar.

Age plays an important role in cognitive decline and swallowing disorders. Sarcopenia, which is an age‐related decrease in both muscle mass and quality, is a contributing factor in dysphagia. As people age, the muscles used for digestion have been shown to be affected negatively (Molfenter et al., 2019). In addition, it has been demonstrated that swallowing dysfunction starts early in Alzheimer's‐type dementia (Humbert et al., 2010). This is the reason why age, EAT‐10, and EdFED show a close association in the subcluster in our study.

Education status and cognitive function are in the same subcluster. Neuropathologic correlations support this notion, indicating that those with a greater cognitive reserve, as measured by years of education, are better able to cope with AD brain pathology without noticeable cognitive losses (Roe et al., 2007). In our analysis, we found that the control group's variable clustering was similarly associated. In other words, clustering does not happen in the absence of diseases. This result clearly illustrates that the variables clustered separately in the neurological diseases.

In their study, Jo et al. (2017) discovered a substantial correlation between swallowing performance and subsets of cognitive processes connected to visual cues, as opposed to functions requiring auditory attention or verbal memory function. Our results were in line with their findings, and in swallowing assessment, cognition should also be taken into consideration. Although memory dysfunction is among the first noticeable symptoms of AD, our findings suggest that swallowing function also shows compromise early on. As the cognition starts to deteriorate, so does the swallowing performance.

As neurogenic dysphagia can reduce oral feeding, cause malnutrition, dehydration, eating improper substances or amounts of food, trouble transferring meals from plate to mouth, difficulties with mastication or swallowing, and cause aspiration, all of which dramatically lessens patients' quality of life and can even prove to be fatal in some cases (Ortega et al., 2017). Dysphagia is frequently neglected and underdiagnosed in vulnerable patient populations, despite its high frequency among the elderly.

As for a clinical implantation of our study, we found that clinicians should be aware of age, education, and cognitive abilities, while working with MND. They should interpret test scores as a whole since they show a close relation in HCA. Recognizing that a patient with neurodegenerative diseases is having eating difficulties and early intervention can give the patient a better chance of maintaining independence.

With regard to another important clinical implication, scores of MMASA and MoCA and education status are closely related as described in figures. Therefore, clinicians should interpret MMASA scores much more carefully since its results can be affected by cognitive impairments. MMASA includes oral‐motor behavior assessment unlike EAT‐10 and EdFED. Dysphagia can be linked to anxiety and depression either because of the severity and effects of the symptoms or as a result of the root cause (Khayyat et al., 2023). When evaluating patients with MND, other factors including depression and anxiety should be taken into consideration.

This study has a number of limitations. Groups were not matched for age distribution. Future research must include proportional distribution, larger patient populations, and subgroups. Additionally, to have a deeper understanding more patient populations could be investigated in future research. In our study, we did not perform instrumental assessment for our patients. It might give more insight if patients having swallowing disorder complaints can be instrumentally evaluated.

It is clear that HCA attempts to group topics with similar perspectives. According to the results of the HCA, EAT‐10, and EdFED yield comparable results regarding swallowing disorders in patients. MMASA, which should also provide information regarding swallowing performance, is surprisingly not in the same cluster. The clustering of MMASA scores and cognitive tests demonstrates that the cognitive abilities of patients play a significant role in determining their swallowing performance. EAT‐10 and EdFED should be used instead of MMASA when evaluating swallowing performance in patients with deteriorating cognitive function. It is strongly advised that clinicians consider these facts when evaluating patients. It is better for a patient to be evaluated with an objective, instrumented test based on the MMASA score in order for the results to be more accurate. This study demonstrates a successful implementation of cluster analysis for patients with various dementia types, which can lead to dysphagia. As a result of our research, we can see that the close relationships between the certain variables in the clusters we got match what the literature points out. In this way, the dendrogram's visual results and clusters made it possible to present the variables in a regular and systematic way.

## AUTHOR CONTRIBUTIONS

**Samet Tosun**: Conceptualization; investigation; writing—original draft; methodology; validation; visualization; writing—review and editing; software; formal analysis; project administration; resources; data curation; supervision. **Fenise Selin Karali**: Conceptualization; investigation; writing—original draft; writing—review and editing; visualization; validation; methodology; software; formal analysis; project administration; resources; supervision; data curation. **Dilber Kacar Kutukcu**: Writing—original draft; investigation; conceptualization; methodology; validation; visualization; writing—review and editing; project administration; formal analysis; software; data curation; supervision; resources. **Nilgün Cinar**: Data curation; resources; supervision; project administration; formal analysis; software; methodology; validation; visualization; writing—review and editing; writing—original draft; investigation; conceptualization. **Sude Kendirli**: Conceptualization; investigation; writing—original draft; methodology; validation; visualization; writing—review and editing; project administration; formal analysis; software; data curation; supervision; resources. **Meltem Sen Aksut**: Data curation; supervision; resources; project administration; software; formal analysis; methodology; validation; visualization; writing—review and editing; investigation; conceptualization; writing—original draft. **Ilayda Albayrak**: Conceptualization; investigation; writing—original draft; methodology; validation; visualization; writing—review and editing; project administration; formal analysis; software; data curation; supervision; resources. **Yusuf Celik**: Conceptualization; investigation; writing—original draft; methodology; validation; writing—review and editing; visualization; project administration; formal analysis; software; data curation; supervision; resources.

## FUNDING INFORMATION

Open access funding provided by the Scientific and Technological Research Council of Türkiye (TÜBİTAK).

## CONFLICT OF INTEREST STATEMENT

The authors declare no conflicts of interest.

### PEER REVIEWS

The peer review history for this article is available at https://publons.com/publon/10.1002/brb3.70005.

## Data Availability

The data that support the findings of this study are available from the corresponding author upon reasonable request.
